
JOURNAL INFORMATION
==============================
NLM Title Abbreviation: Behav Neurol
Journal ID: Behav Neurol
NLM Journal ID: 8914585
Journal ID: BN

Behavioural Neurology

ISSN: 0953-4180
EISSN: 1875-8584
Publisher: Wiley

ARTICLE INFORMATION
==============================
PMCID: PMC5434336
PMID: 21422546
DOI: 10.3233/BEN-2010-0286
Article ID: 230506
Article version: 1
Subjects: Research Article

Long-Term Retention Deficit Involvement in Prodromal Alzheimer’s Disease

Jonin P. Y. *
Vichard H.
Leray E.
Sarazin M.
Dubois B.
Belliard S.
PréAl study group
CHU PONTCHAILLOU CMRR Haute-Bretagne & Service de Neurologie Rennes France
*P. Y. Jonin: pierreyves.jonin@chu-rennes.fr
Print publication date: 2010
Electronic publication date: 2011 Mar 7
Volume: 23
Issue: 4
First page: 169
Last page: 171
Received 2011 Jan 31; Accepted 2011 Jan 31
Copyright: Copyright © 2010 Hindawi Publishing Corporation and the authors.
Copyright year: 2010
License: This is an open access article distributed under the Creative Commons Attribution License, which permits unrestricted use, distribution, and reproduction in any medium, provided the original work is properly cited.
License URL: https://creativecommons.org/licenses/by/3.0/

==============================
Behavioural Neurology 23 (2010) 169–171
DOI 10.3233/BEN-2010-0286
IOS Press

### Long-term retention deficit involvement in prodromal Alzheimer's disease

P.Y. Jonin*, H. Vichard, E. Leray, M. Sarazin, B. Dubois, S. Belliard and PréAl study group
*CHU PONTCHAILLOU, CMRR Haute-Bretagne & Service de Neurologie, Rennes, France*

### 1. Introduction

Since episodic memory impairments have been pointed out as the first symptom to occur in Alzheimer's disease (AD), what have we learned about the nature of those impairments?

There is still no consensus regarding the cognitive substrates of the inaugural memory disorder. Encoding, storage as well as retrieval or recognition had all been pointed out, but a compound deficit has also been suggested [1]. We can point out several reasons. First, research has mainly focused on memory measures that could predict incident dementia, therefore study designs generally do not allow inference about the cognitive processes engaged in the tasks used (e.g. [2]). Secondly, concepts of encoding, storage, retrieval are quite rarely defined in literature, leading to conceptual and operational overlaps between so-called "learning, acquisition, encoding, storage, consolidation, etc.".

Nonetheless, given the importance of memory deficit for diagnosis, a detailed knowledge of underlying cognitive features of the memory deficit is required [3]. How to improve such process dissociation?

The first method consists in the development of specific paradigms. Free and Cued Selective Reminding Test [4] was developed to target genuine memory deficits in dementia, and it is the most popular screening tool in French memory clinics. Unfortunately, studies using FCSRT did not achieve any clear consensus. Noteworthy, coordination between encoding and retrieval seems to enhance specificity of the task for incident dementia.

The second method relies on a qualitative, item-by-item analysis of learning performance. Pioneer studies of Endel Tulving [5] and Buschke and Altman [6] achieved an operational distinction between long- and short-term processes by examining the fate of each item between learning trials. A recent study [7] used this approach within a cross-sectional design study, which suggested a deficit in acquisition and retention processes in early AD as well as in "MCI" participants. Unfortunately, cross-sectional data do not allow to draw conclusions about the status of MCI group. Moreover, their "Gained Access" measure (items that occur at trial n but not at trial n-1) could mirror working memory processes rather than the long-term effect that authors wished to assess.

We therefore aimed at differentiate which process is precociously impaired before dementia occurs. To fill-in preceding methodological caveats, we wished to use a qualitative, intra-individual analysis with a learning task that allows encoding-retrieval coordination. Finally, clear definitions of measured process were required.

### 2. Methods

### 2.1. Participants

"PréAl" study [8] was a 3-years longitudinal prospective study involving 14 expert memory centres. Participants were included on the basis of (1) a subjective memory complaint; (2) an objective cognitive impairment (one or more word missing at MMSE recall and / or a score $<=$ 29 at Isaac set test); (3) preserved global cognitive functioning (MMSE between 25 and

*Corresponding author: P.Y. Jonin, CHU PONTCHAILLOU, CMRR Haute-Bretagne & Service de Neurologie 2, rue H. Le Guilloux, 35033 Rennes Cx 9, France. E-mail: Pierreyves.jonin@chu-rennes.fr.

ISSN 0953-4180/10/$27.50 © 2010 – IOS Press and the authors. All rights reserved

29); (4) normal score ore only one item impaired at the first level in the four IADL and (5) the absence of dementia according to DSM-III r criteria.

At the beginning of 2010, we contacted each participant centre to update "stable group" status, almost 7 years after the end of PréAl study. Inclusion criteria were: (1) one year or more follow-up after PréAl; (2) complete neurological and neuropsychological examination available; (3) AD cases diagnosed by expert centers according to NINCDS-ADRDA criteria for probable AD. Incident AD group was further classified in 2 subgroups depending on the delay before diagnosis : Early-onset ($<$ 24 months) *vs.* Late-onset ($\geqslant$ 24 months). For the purpose of the present study, we added a group of 23 control subjects.

### 2.2. Procedure

Only baseline data from Free and Cued Selective Reminding Test was further considered. Following indexes were computed:

*Acquisition Index* (*LT A*)*:* proportion of items that occur for the first time (either free or cued recall) after a delay. *Acquisition index* therefore corresponds to acquisition in long-term store as a product of information processing into a stable mnemonic representation.

*Retention Index* (*LT R*)*:* proportion of items that occur at trial $n + 1$ among items given at trial $n$ (Free or Cued Recall). *Retention index* therefore measures the maintenance in long-term store by associations with other stable representations.

### 3. Results

Among 279 participants initially screened, 192 fulfilled inclusion criteria, including 33 incidents AD and 159 free of dementia at PréAl follow-up. 14 experts centres were contacted for N $=$ 159 stable participants. 7 centres answered covering 70 participants, resulting in the final inclusion of 27 stable participants and 36 incident AD. One-way ANOVAs failed to show any difference between groups for Age and Education, and sex ratio was virtually identical in each group. MMSE total score differed between groups, post-hoc analysis showing that all comparisons were significant: controls $>$ stable $>$ late-onset AD $>$ early-onset AD.

Groups differed regarding LT A and LT R indexes, and post-hoc analysis yielded the same pattern for both indexes: Controls $=$ Stable $>$ late-onset AD $>$ early-onset AD (see Table). ROC curves showed that both indexes accurately discriminated incident AD from controls, with similar sensitivity/specificity (83%–100%) for the comparison [controls vs. early-onset AD]. However, LT Retention achieved a slightly better sensitivity/specificity (77%–78% for LT A vs. 83%–87% for LT R) for the comparison [controls vs. late-onset AD]. Furthermore, delay before diagnosis only correlated with LT R ($r = 0.412$; $p = 0.01$).

### 4. Discussion

Our data speak for the precocious impairment of long-term acquisition and retention more than 3 years before AD dementia can currently be diagnosed. Those results fit well with the main relevant study in the field [7], but they also extend its findings. Indeed, we are not aware of a previous longitudinal study involving the combination of a qualitative analysis with a learning paradigm allowing for encoding specificity. This approach avoids short-term memory effects as well as artefact retrieval deficits due to cognitive aging. Moreover, our study suggests that long-term retention may play a prominent role in the memory disorder inaugurating AD. Its better sensitivity and specificity for predicting late-onset AD indicates that this process could be the first impaired. Its correlation with delay before diagnosis furthermore suggests that it is associated with the evolution of the underlying pathology.

Other studies failed to show any long-term retention deficit in prodromal phase of AD. We argue that two main reasons can be pointed out. First, use of quantitative, inter-individual analysis of performances such as saving score method do not allow to discriminate between short- and long-term acquisition or storage processes [9]. Secondly, measuring long-term retention implies to control for items to be encoded in long-term store. This is usually not the case in previous reports. Thus, Perri et al. [10] argued that their finding of an increased forgetting rate in MCI participants was not due to a real long-term retention deficit but rather to an inadequate transfer from short-term to long-term store. However, authors used a word-list classical paradigm and compared delayed recall with immediate recall, without correcting the forgetting score for the items really acquired in long-term store. At variance with this interpretation, our data clearly speak for a precocious and independent involvement of long-term retention in prodromal AD.

Table 1
Statistics for clinical & experimental variables. LT = Long-Term. Except for sex ratio, one-way analysis of variance were performed. Welsh and Brown-Forsythe tests were used to confirm ANOVAs when appropriate (i.e. unequal homogeneity of variance)

| | Controls N = 23 | Stable Cog. Impairment N = 27 | Late-onset AD N = 18 | Early -onset AD N = 18 | F / Chi² | p | post-hoc |
|---|---|---|---|---|---|---|---|
| *Age* | 73,2 *(7,4)* | 70,0 *(5,3)* | 72,3 *(5,7)* | 74,3 *(4,8)* | 2,285 | ***0,085*** | |
| *Ratio M : F* | 7 : 16 | 13 : 14 | 11 : 7 | 8 : 10 | 3,960 | ***0,266*** | |
| *Education* | 2,5 *(0,8)* | 3,0 *(0,9)* | 3,1 *(0,9)* | 3,0 *(0,8)* | 2,074 | ***0,110*** | |
| *MMSE* | 29,2 *(1,2)* | 27,9 *(1,1)* | 27,4 *(1,0)* | 26,9 *(0,8)* | 18,496 | ***<0,01*** | Controls > Stable > Early diag.AD ; Stable = Lately Diag.AD |
| *Mean follow-up (months)* | | 83,4 *(16,7)* | 39,8 *(17,0)* | 13,6 *(4,7)* | | | |
| *LT Acquisition* | 93,5 *(6,3)* | 90,7 *(10,4)* | 76,2 *(12,1)* | 65,2 *(15,8)* | 25,791 | ***<0,01*** | Controls = Stable > Lately Diag.AD > Early diag.AD |
| *LT Retention* | 98,5 *(2,5)* | 96,8 *(3,7)* | 88,8 *(10,4)* | 81,5 *(11,2)* | 23,425 | ***<0,01*** | Controls = Stable > Lately Diag.AD > Early diag.AD |

### References

[1] F. Lekeu and M. Van Der Linden, *Le fonctionnement de la mémoire épisodique dans la maladie d'Alzheimer*, *In* Les troubles de la mémoire dans la maladie d'Alzheimer, Ed. Solal, Marseille, 2005, pp. 73–117.

[2] M.F. Elias, A. Beiser, P.A. Wolf, R. Au, R.F. White and R.B. D'Agostino, The Preclinical Phase of Alzheimer's disease. A 22-Year prospective study of the Framingham cohort, *Archives of Neurology* **57** (2000), 808813.

[3] D. Mungas, L. Beckett, D. Harvey, S. Tomaszewski Farias, B. Reed, O. Carmichael al., Heterogeneity of Cognitive Trajectories in Diverse Older Persons, *Psychology and Aging* (2 August 2010), 1–14.

[4] H. Grober and H. Buschke, Genuine memory deficits in dementia, *Developmental Neuropsychology* **3**(1) (1987), 13–36.

[5] E. Tulving, Intratrial and Intertrial retention: notes toward a theory of free recall verbal learning, *Psychology Review* **71**(3) (1964), 219–236.

[6] H. Bushke and P.F. Altman, Evaluating storage, retention, and retrieval in disordered memory and learning, *Neurology* **24** (1974), 1019–1025.

[7] C.J.A. Moulin, N. James, J.E. Freeman and R.W. Jones, Deficient acquisition and consolidation : intertrial free recall performance in Alzheimer's disease and Mild Cognitive Impairment, *Journal of Clinical and Experimental Neuropsychology* **26**(1) (2004), 1–10.

[8] M. Sarazin, C. Berr, J. De Rotrou, C. Fabrigoule, F. Pasquier, S. Legrain al., Amnestic syndrome of medial temporal type identifies prodromal Alzheimer's disease; A longitudinal study, *Neurology 6* **69**(19) (2007), 1859–1867.

[9] Y.L. Chang, M.W. Bondi, C. Fennema-Notestine, L.K. McEvoy, D.J. Hagler, Jr. and M.W. Jacobson, Brain substrates of learning and retention in Mild Cognitive Impairment diagnosis and progression to Alzheimer's disease, *Neuropsychologia* **48** (2010), 1237–1247.

[10] R. Perri, L. Serra, G.A. Carlesimo and C. Caltagirone, Amnestic Mild Cognitive Impairment : different memory profiles in subjects who converted or did not to Alzheimer's disease, *Neuropsychology* **21**(5) (2007), 549–558.
